# Circulating tumor cells as prognostic and predictive markers in metastatic breast cancer patients receiving first-line systemic treatment

**DOI:** 10.1186/bcr2907

**Published:** 2011-06-15

**Authors:** Mario Giuliano, Antonio Giordano, Summer Jackson, Kenneth R Hess, Ugo De Giorgi, Michal Mego, Beverly C Handy, Naoto T Ueno, Ricardo H Alvarez, Michelino De Laurentiis, Sabino De Placido, Vicente Valero, Gabriel N Hortobagyi, James M Reuben, Massimo Cristofanilli

**Affiliations:** 1 Department of Hematopathology, The University of Texas MD Anderson Cancer Center, 1515 Holcombe Blvd, Houston, TX 77030, USA; 2 Department of Molecular and Clinical Oncology and Endocrinology, University of Naples Federico II, via Pansini 5, 80131 Naples, Italy; 3 Currently: Breast Center, Baylor College of Medicine, One Baylor Plaza, Houston, TX 77030, USA; 4 Breast Medical Oncology, The University of Texas MD Anderson Cancer Center, 1515 Holcombe Blvd, Houston, TX 77030, USA; 5 Department of Biostatistics, The University of Texas MD Anderson Cancer Center, 1515 Holcombe Blvd, Houston, TX 77030, USA; 6 Currently: Medical Oncology, Istituto Scientifico Romagnolo per lo Studio e la Cura dei Tumori, Via Maroncelli 40, 47014 Meldola (FC), Italy; 7 Currently: Department of Medical Oncology, School of Medicine, Comenius University, Klenova 1, Bratislava 833 10, Slovakia; 8 Laboratory Medicine, The University of Texas MD Anderson Cancer Center, 1515 Holcombe Blvd, Houston, TX 77030, USA; 9 Department of Medical Oncology, Fox Chase Medical Center, 333 Cottman Avenue, Rm 315, Philadelphia, PA 19111-2497, USA

## Abstract

**Introduction:**

Circulating tumor cells (CTCs) represent an independent predictor of outcome in patients with metastatic breast cancer (MBC). We assessed the prognostic impact of CTCs according to different first-line systemic treatments, and explored their potential predictive value in MBC patients.

**Methods:**

We retrospectively evaluated 235 newly diagnosed MBC patients, treated at the University of Texas MD Anderson Cancer Center. All patients had a baseline CTC assessment performed with CellSearch^®^. Progression-free survival and overall survival were compared with the log-rank test between groups, according to CTC count (< 5 vs. ≥ 5) and type of systemic therapy. We further explored the predictive value of baseline CTCs in patients receiving different treatments.

**Results:**

At a median follow-up of 18 months, the CTC count was confirmed to be a robust prognostic marker in the overall population (median progression-free survival 12.0 and 7.0 months for patients with CTC < 5 and ≥ 5, respectively; *P *< 0.001). Conversely, in patients with human epidermal growth factor receptor-2-overexpressed/amplified tumors receiving trastuzumab or lapatinib, the baseline CTC count was not prognostic (median progression-free survival 14.5 months for patients with CTC < 5 and 16.1 months for those with CTC ≥ 5; *P *= 0.947). Furthermore, in patients with human epidermal growth factor receptor-2 normal tumors, a baseline CTC count ≥ 5 identified subjects who derived benefit from more aggressive treatments, including combination chemotherapy and chemotherapy plus bevacizumab.

**Conclusions:**

This analysis suggests that the prognostic information provided by CTC count may be useful in patient stratifications and therapeutic selection, particularly in the group with positive CTCs, in which various therapeutic choices may procure differential palliative benefit.

## Introduction

The prognosis of patients with metastatic breast cancer (MBC) has significantly improved over the last two decades [1]. Despite these advances, metastatic disease remains largely incurable and the main goal of systemic treatment is to prolong survival and maintain a high quality of life [2]. Women with MBC represent a heterogeneous group of patients with different outcomes. Classical factors such as age at diagnosis, hormone receptor status, human epidermal growth factor receptor-2 (HER-2) overexpression/amplification, and site of metastases are currently used to stratify patients into groups with different prognoses and to predict response to systemic treatments [3]. Oncologists choose from a wide variety of standard treatment options, including endocrine therapies, chemotherapy-based regimens and biologically targeted treatments, which may provide differential palliative benefit [4]. The introduction of new anti-tumor agents in clinical practice necessitates the improvement of patient selection for personalized treatment strategies. Indeed, the availability of early predictive markers of treatment response could prevent exposure to ineffective therapies as well as to unnecessary treatment-related toxicity, and possibly reduce the costs of treatment in patients with refractory disease [5].

Recently, the enumeration of circulating tumor cells (CTCs) in the peripheral blood of cancer patients has been associated with both disseminated disease and a higher risk of cancer progression [6]. Several lines of evidence confirm that the detection of CTCs represents a new and reliable tool to predict the outcome of MBC patients [7,8]. Furthermore, the enumeration of CTCs at different time points during treatment has proven to be a reliable surrogate marker of treatment response, and a potential alternative for non-invasive therapy monitoring [9-11]. Among several methods developed for CTC detection, the CellSearch^® ^system (Veridex LLC, Warren, NJ, USA) is the only US Food and Drug Administration-cleared test for CTC enumeration in clinical practice [12]. Nevertheless, the availability of improved and standardized techniques for detection and molecular analysis of CTCs has allowed researchers to better define the unique phenotypic characteristics of these cells and their putative roles in cancer dissemination [13]. As a predictor of disease progression and precursors of metastases, CTCs provide an ideal model for the development of new targeted treatments. Indeed, the unique nature of these cells, which can be genetically different from the primary tumor, is a peculiar feature of tumor biology that should be considered when selecting targeted therapies [14-16].

Despite their potential therapeutic benefit, CTCs have been studied mainly as a prognostic marker, while their value as a predictive factor has remained largely unclear. The objective of our retrospective study was to assess the prognostic value of baseline CTCs in patients receiving different first-line systemic treatments for MBC, and to determine the possible predictive value of this marker.

## Materials and methods

### Study design

We retrospectively evaluated a population of 517 consecutive MBC patients treated at The University of Texas MD Anderson Cancer Center, Houston, TX, USA. Each patient had a standard CTC assessment before starting systemic treatment. From all the patients examined, we selected 235 women who received first-line systemic therapy between September 2004 and November 2009. The principal eligibility criteria for this study included patients with newly diagnosed metastatic disease, CTC evaluation performed as standard of care within 30 days before starting any systemic treatment, and availability of treatment and follow-up information. Tumor response was evaluated according to the response evaluation criteria in solid tumors [17,18]. The histological type, tumor grade, hormone receptor status, and HER-2/neu status were evaluated on the primary tumor or, when available, on metastatic disease. The HER-2/neu status was determined using immunohistochemistry and/or fluorescent *in situ *hybridization techniques. Patients' treatments were selected according to the National Comprehensive Cancer Network and Institutional guidelines [19]. The institutional review board at the University of Texas MD Anderson Cancer Center approved the study and granted a waiver of informed consent, considering the retrospective nature of the study (DR10-0227).

### Isolation and enumeration of circulating tumor cells

Blood samples (7.5 ml) were drawn into CellSave^® ^tubes (Veridex LLC), which were maintained at room temperature and processed within 72 hours of collection. The standardized CellSearch System^® ^(Veridex LLC) was used for isolating and enumerating CTCs, as reported previously [7,14]. CTCs were defined as nucleated EpCAM-positive cells, lacking CD45 but expressing cytoplasmic cytokeratins 8, 18, and 19. All CTC evaluations were performed by qualified and trained personnel. Patients were categorized according to baseline CTC counts as having favorable (< 5 CTCs/7.5 ml blood) or unfavorable (≥ 5 CTCs/7.5 ml blood) outcome.

### Statistical analysis

All clinical data were collected independently by two physicians (MG and AG) from the MD Anderson electronic medical record (ClinicStation^®^). Progression-free survival (PFS) was calculated from the date of CTC evaluation to the date of clinical disease progression or death; in the absence or either progression or death, patients were censored at the date of the last follow-up. Overall survival (OS) was defined as the time elapsed between the date of CTC assessment and the date of either death or last follow-up.

The Fisher's exact test and the Pearson's chi-square test were used to determine significant differences in patient characteristics according to baseline CTC count. The prognostic effect of CTCs was explored in the overall population and within different subgroups of patients according to treatment received (endocrine treatment or different regimens of chemotherapy). The PFS and OS were estimated using the Kaplan-Meier product limit method. The log-rank test was used to compare PFS and OS between groups, according to the CTC count (< 5 vs. ≥ 5) and the type of systemic therapy. To confirm CTCs as an independent prognostic factor, Cox proportional hazard models for PFS and OS were fit, adjusting for hormone receptor status (positive vs. negative), HER-2/neu status (amplified/overexpressed vs. normal), number of metastatic sites (1 vs. 2 vs. ≥ 3) and site of metastases (visceral vs. other).

The predictive value of CTCs was explored by evaluating the interaction between efficacy of different treatments and baseline value of CTCs. The effect of treatments was expressed as hazard ratios with 95% confidence intervals (CIs), and a forest plot was generated to display results. To evaluate the interaction between treatment effect and CTC count, we quantified the heterogeneity between subgroups (CTCs < 5 and ≥ 5) with the Higgins' I^2 ^index [20]. All statistical analyses, performed using the PASW Statistical Analysis for Social Sciences statistics 18 software (SPSS Inc., Chicago, IL, USA), were two-sided and *P *< 0.05 was considered statistically significant.

## Results

### Patient characteristics

Demographic and disease characteristics of the 235 patients evaluated in this analysis are reported in Table 1. The median age of patients was 53 years (range 28 to 82 years). One hundred and fifty-one patients had hormone receptor-positive disease. Visceral metastases were detected in approximately 60% of patients. The baseline value of CTCs per 7.5 ml blood was < 5 in 141 (60%) and ≥ 5 in 94 (40%) patients. A higher percentage of patients with ≥ 5 CTCs at baseline had three or more metastatic sites of disease at baseline (45.7% vs. 29.8%, *P *= 0.011). No statistically significant difference in metastatic site (visceral vs. other) or in the distribution of immunohistochemistry-defined molecular subtypes was observed according to CTC value.

**Table 1:** Patient characteristics stratified by baseline circulating tumor cell value

Variable	Overall	CTC < 5	CTC ≥ 5	*P *value
All patients	235 (100)	141 (60)	94 (40)	-
Age (years)	53 (23 to 82)	53 (28 to 82)	53 (23 to 81)	0.451
Follow-up (months)	18 (1 to 65)	20 (1 to 65)	18 (3 to 61)	-
HR^+^/HER-2 normal	130 (55.3)	74 (52.5)	56 (59.6)	0.349
HR^+^/HER-2 overexpressed/amplified	21 (8.9)	15 (10.6)	6 (6.4)	0.352
HR^-^/HER-2 overexpressed/amplified	22 (9.4)	13 (9.2)	9 (9.6)	1.0
Triple receptor negative	62 (26.4)	39 (27.7)	23 (24.4)	0.651
Visceral metastases	140 (59.6)	80 (56.7)	60 (63.8)	0.342
Number of metastatic sites				
1	85 (36.2)	61 (43.3)	24 (25.5)	0.011*
2	65 (27.6)	38 (26.9)	27 (28.8)	
≥ 3	85 (36.2)	42 (29.8)	43 (45.7)	

Data presented as *n *(%) or median (range). CTC, circulating tumor cell; HER-2, human epidermal growth factor receptor-2; HR^+^, estrogen receptor-positive and/or progesterone receptor-positive; HR^-^, both estrogen receptor-negative and progesterone receptor-negative. * Statistical significant value.

All patients received first-line systemic therapy for newly diagnosed MBC (Table 2). Forty-seven patients (20%) received endocrine therapy, 109 (46.4%) were treated with chemotherapy alone, 39 (16.6%) received bevacizumab associated with taxane-based chemotherapy, and 40 patients (17%) with HER-2-overexpressed/amplified disease received chemotherapy combined with a HER-2 targeting drug, including trastuzumab and lapatinib.

**Table 2:** Treatment administered

Treatment	Age (years)	Overall	CTC < 5	CTC ≥ 5
Endocrine treatment	56 (36 to 82)	47 (20.0)	33 (70.2)	14 (29.8)
Aromatase inhibitor		35 (74.5)	25 (71.4)	10 (28.6)
Tamoxifen		8 (17.0)	6 (75.0)	2 (25.0)
Fulvestrant		4 (8.5)	2 (50.0)	2 (50.0)
Monochemotherapy	57 (31 to 81)	45 (19.1)	23 (51.1)	22 (48.9)
Taxane		21 (46.7)	12 (57.1)	9 (42.9)
Other^a^		24 (53.3)	11 (45.8)	13 (54.2)
Combination chemotherapy	53 (23 to 78)	64 (27.2)	40 (62.5)	24 (37.5)
Taxane + anthracycline		25 (39.1)	16 (64.0)	9 (36.0)
Taxane + capecitabine		22 (34.4)	13 (59.1)	9 (40.9)
Taxane + other cytotoxic agent^b^		7 (10.9)	5 (71.4)	2 (28.6)
Other^c^		10 (15.6)	6 (60.0)	4 (40.0)
Chemotherapy + anti-HER2 drugs	53 (28 to 81)	40 (17.0)	26 (65.0)	14 (35.0)
Chemotherapy + trastuzumab^d^		30 (75.0)	17 (57)	13 (43)
Chemotherapy + lapatinib		10 (25.0)	9 (90)	1 (10)
Chemotherapy + bevacizumab	49 (30 to 67)	39 (16.6)	19 (48.7)	20 (51.3)
Monochemotherapy + bevacizumab		32 (82.1)	18 (56.2)	14 (43.8)
Polychemotherapy + bevacizumab		7 (17.9)	1 (14.3)	6 (85.7)

Data presented as *n *(%) or median (range). CTC, circulating tumor cell; HER-2, human epidermal growth factor receptor-2. ^a^Capecitabine (*n *= 17), epirubicin (*n *= 1), vinorelbine (*n *= 1), gemcitabine (*n *= 3), carboplatin (*n *= 2). ^b^Taxane + carboplatin (*n *= 6), taxane + gemcitabine (*n *= 1). ^c^5-Fluorouracil + epirubicin + cyclophosphamid (*n *= 5), fluorouracil + doxorubicin + cyclophosphamid (*n *= 2), gemcitabine + carboplatin (*n *= 3). ^d^Taxane-based chemotherapy + trastuzumab (*n *= 24).

### Prognostic value of circulating tumor cells in the overall population

**Figure 1:**
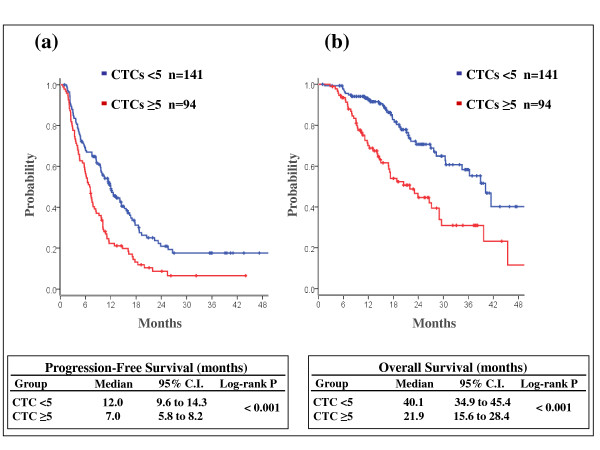
**Prognostic value of circulating tumor cells in the overall population**. Estimated **(a) **progression-free survival and **(b) **overall survival according to baseline circulating tumor cell (CTC) value (< 5 vs. ≥ 5) in the overall population. C.I., confidence interval.

The median follow-up for all patients and for patients still alive was 17 months and 18 months, respectively. At the time of the analysis, 87 patients (37%) had died and 179 patients (76%) experienced disease progression. We found a remarkable correlation between the baseline value of CTCs and the outcome of all patients. The median PFS was 12.0 months (95% CI = 9.6 to 14.3) for patients with CTCs < 5 and 7.0 months (95% CI = 5.8 to 8.1) for those with CTCs ≥ 5 (log-rank *P *< 0.001). The median OS was 40.1 months (95% CI = 34.9 to 45.4) for women with a low CTC and 21.9 months (95% CI = 15.5 to 28.3) for those with CTCs ≥ 5 (log-rank *P *< 0.001; Figure 1).

Furthermore, in multivariate analysis the baseline count of CTCs was confirmed to be an independent predictor of PFS and OS, regardless of hormone receptor status, HER-2 status, location or number of metastatic sites (Table 3).

**Table 3:** Multivariable Cox proportional hazards model

Variable	Progression-free survival		Overall survival	
	**Hazard ratio (95% CI)**	***P *value**	**Hazard ratio (95% CI)**	***P *value**

HR^+ ^vs. HR^-^	0.63 (0.46 to 0.86)	0.003	0.51 (0.34 to 0.79)	0.002
HER-2 amplified/overexpressed vs. HER-2 normal	0.52 (0.34 to 0.79)	0.002	0.39 (0.21 to 0.73)	0.003
Visceral vs. other metastases	1.39 (0.92 to 2.05)	0.095	1.77 (0.95 to 3.30)	0.074
Number of metastatic sites (1 vs. 3 vs. ≥ 3)	0.88 (0.70 to 1.10)	0.266	0.67 (0.47 to 0.95)	0.024
Circulating tumor cells (< 5 versus ≥ 5)	0.58 (0.43 to 0.79)	< 0.001	0.40 (0.26 to 0.62)	< 0.001

CI, confidence interval; HER-2, human epidermal growth factor receptor-2; HR^+^, estrogen receptor-positive and/or progesterone receptor-positive; HR^-^, both estrogen receptor-positive and progesterone receptor-negative.

### Effect of different treatments on the circulating tumor cell detection rate

We analyzed the effect of different systemic treatments on CTC count. For 144 patients (61%), a follow-up evaluation of CTCs was available. The median time between baseline and follow-up CTC evaluations was 10 weeks (15% of the patients had a follow-up count within week 6 from baseline, 53% from week 6 to week 12, 22% from week 13 to week 19, and 10% from week 20 to week 45). The effect of chemotherapy plus bevacizumab and chemotherapy plus HER-2-targeting drugs in patients with a high baseline CTC count was considerable, with a reduction of CTC number to below the threshold of 5 in 16 out of 17 (94%) and in nine out of nine (100%) subjects, respectively. Instead, chemotherapy or endocrine therapy were associated with a CTC reduction in 16 out of 40 cases (40%). Specifically, endocrine treatment was able to reduce CTCs under the threshold of 5 only in one out of 10 (10%) patients - whereas chemotherapy alone had a more pronounced effect, inducing a reduction of CTCs to < 5 in 15 out of 30 cases (50%).

### Prognostic value of circulating tumor cells according to different first-line treatments

**Figure 2:**
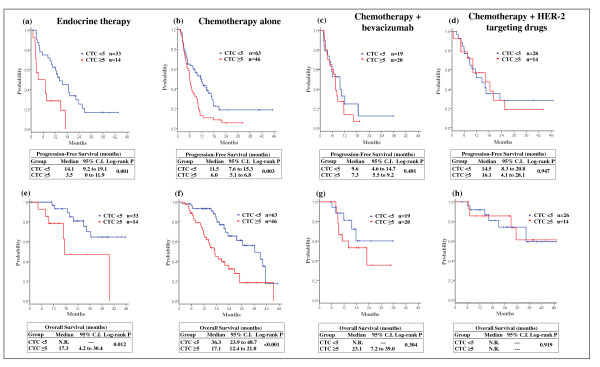
**Prognostic value of circulating tumor cells according with different first-line treatments**. Estimated progression-free survival and overall survival according to baseline circulating tumor cell (CTC) value (< 5 vs. ≥ 5) in patients receiving **(a), (e) **endocrine therapy, **(b), (f) **chemotherapy alone, **(c), (g) **chemotherapy + bevacizumab, and **(d), (h) **chemotherapy + human epidermal growth factor receptor-2 (HER-2)-targeting drugs. C.I., confidence interval.

The differential ability of each modality of treatment to reduce the CTC number led us to evaluate whether the most effective therapies could impact the negative prognostic value associated with a high count of CTCs. We evaluated the CTC prognostic value in all treatment groups, including endocrine therapy, chemotherapy alone, chemotherapy plus bevacizumab, and chemotherapy plus HER-2-targeting drugs (Figure 2).

CTCs remained a strong prognostic indicator in patients receiving endocrine treatment or chemotherapy alone. In both of these treatment groups, a high CTC count was associated with poor outcome, particularly in patients receiving endocrine therapy (median PFS 14.1 vs. 3.5 months for subjects with CTC < 5 and ≥ 5, respectively; log-rank *P *= 0.001; Figure 2). Even when considering only the most effective chemotherapy regimens, including either a taxane as a single agent or in combination with other chemotherapeutic agents (that is, anthracyclines, capecitabine, gemcitabine, carboplatin), chemotherapy administered without any biologically targeted agent did not impact the negative prognostic value of CTCs (median PFS 12.6 months for patients with CTCs < 5 and 7.1 for those with CTCs ≥ 5, *P *= 0.03). Conversely, in patients with HER-2-overexpressed/amplified disease treated with an anti-HER-2-based treatment (trastuzumab *n *= 30, lapatinib *n *= 10), the prognostic value of CTCs was no longer sustainable as subjects with baseline CTCs ≥ 5 received a dramatic survival benefit from this therapy (median PFS 16.1 months, 95% CI = 4.1 to 28.1 months; Figure 2). Furthermore, in women receiving taxane-based chemotherapy plus bevacizumab, CTCs < 5 were associated with neither a statistically significantly longer PFS nor OS in comparison with CTCs ≥ 5 (median PFS 9.6 months and 7.3 months for patients with CTCs < 5 and ≥ 5, respectively, *P *= 0.481; Figure 2), suggesting a therapeutic benefit confined to the worse prognostic group.

### Predictive value of circulating tumor cells

Of the 148 patients with HER-2 normal disease who were treated with chemotherapy, 64 (43.2%) received combination chemotherapy, 45 (30.4%) received single-agent chemotherapy, and 39 (26.4%) were treated with chemotherapy plus bevacizumab (Table 2). Those treatments were selected according to patient characteristics (such as age, co-morbidity) and to the traditional predictive markers in use at the time of therapy administration.

**Figure 3:**
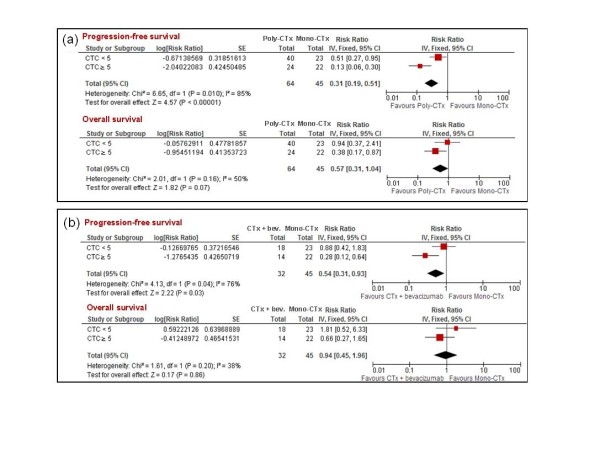
**Predictive value of circulating tumor cells**. Comparison of different first-line treatments according to circulating tumor cell (CTC) baseline value. **(a) **Combination chemotherapy (poly-CTx) versus single-agent chemotherapy (mono-CTx). **(b) **Monochemotherapy + bevacizumab (CTx + bev.) versus single-agent chemotherapy (mono-CTx). CI, confidence interval; IV, inverse variance; SE, standard error.

We sought to explore a hypothetical predictive value for CTCs, comparing different treatments (combination chemotherapy versus monochemotherapy, and monochemotherapy plus bevacizumab vs. monochemotherapy alone) in patients with low (< 5) or high (≥ 5) baseline CTC counts. Combination chemotherapy was superior to single-agent chemotherapy, in terms of PFS, in both CTC groups, although the benefit provided by combination regimens was primarily confined to patients with CTCs ≥ 5 (test for heterogeneity *P *= 0.01; Figure 3). With respect to OS, combination chemotherapy was superior to monochemotherapy only in patients with CTCs ≥ 5, but the heterogeneity between the two subgroups was not statistically significant (test for heterogeneity *P *= 0.16; Figure 3). Furthermore, the association of chemotherapy with bevacizumab was superior to monochemotherapy, regarding PFS, but only in patients with a high baseline CTC count (hazard ratio = 0.88, 95% CI = 0.42 to 1.83 in patients with CTCs < 5; and hazard ratio = 0.28, 95% CI = 0.12 to 0.64, in those with CTCs ≥ 5; test for heterogeneity *P *= 0.04; Figure 3).

## Discussion

Our analysis confirms that CTCs are a clinically valuable and independent prognostic marker in newly-diagnosed MBC patients. Indeed, in the overall population, baseline counts of CTCs were able to discriminate between two groups of patients with different outcome, independently of traditional prognostic factors (hormone receptor, HER-2, or spread of metastatic disease). Moreover, our data showed that the various first-line treatment modalities may have differing capabilities in reducing the number of CTCs. Based on these results, we hypothesized that each treatment can provide a different survival benefit in patients with a high baseline CTC count. Our data demonstrated that women with high baseline CTC counts received very little survival benefit from first-line endocrine treatment, even if they were appropriate candidates for this therapy based on the hormone receptor status of their primary or metastatic tumor. Despite the limited statistical power of this analysis, our findings suggest that endocrine therapy might be inadequate as first-line treatment for MBC patients with a high number of CTCs and alternative approaches should be prospectively tested for this population. Moreover, in patients receiving chemotherapy alone, high baseline CTC counts identified subjects who received a small benefit from systemic treatment and would undoubtedly be destined to early progression of disease and shorter overall survival.

Interestingly, we found a lack of prognostic significance of baseline CTC counts in patients receiving chemotherapy along with targeted treatments. HER-2-targeting drugs combined with chemotherapy reduced the number of CTCs in all patients with a high baseline CTC count. Our findings confirm previous preclinical and clinical studies demonstrating a selective action of trastuzumab against CTCs [21-23]. The ability of trastuzumab to target CTCs, probably acting through antibody-dependent cell-mediated cytotoxicity, could explain the high survival rates exhibited in our study by patients with HER-2-positive disease, who had baseline CTCs ≥ 5. In a recent study, however, Riethdorf and colleagues showed that neoadjuvant trastuzumab-based treatment, in a cohort of patients with HER-2-positive early and locally advanced breast cancer, had a limited effect on the number of HER-2-overexpressing CTCs [14]. On the contrary, it was recently shown that trastuzumab is capable of reducing CTC-expressing HER-2 even in patients with HER-2 normal tumors, suggesting another potential therapeutic use for this agent [24]. There are currently no reports on the effect of lapatinib on CTCs, and future in-depth studies should evaluate the potentially differential effect of the HER-2-targeted therapies on CTCs.

Interestingly, bevacizumab administered in combination with taxane-based chemotherapy reduced the number of CTCs in almost all patients who began therapy with a high count. This reduction did not, however, translate into a striking survival benefit. Indeed, among women receiving bevacizumab, there was still a trend in survival favoring patients with CTCs < 5, although the difference was not statistically significant. This result is consistent with previously published data showing an unclear prognostic impact of CTCs in patients receiving bevacizumab. Bidard and colleagues have shown that baseline CTCs predicted worse time to progression in 67 breast cancer patients receiving bevacizumab-based therapy, only using a cut-off point of three CTCs, with no clinical significance associated with the traditional threshold [25]. A recently published meta-analysis of 43 randomized trials, comparing combination chemotherapy versus single-agent chemotherapy regimens, showed a statistically significant advantage in terms of survival, tumor response and time to progression for polychemotherapy, although it caused more toxicity [26]. An alternative to polychemotherapy for HER-2-negative patients has been provided by the association of bevacizumab with chemotherapy. Several randomized trials - including E2100, AVADO, RIBBON-1 and RIBBON-2 - have demonstrated that bevacizumab-based combination therapies, compared with chemotherapy alone, improved the response rate and PFS, although no study showed an improvement in OS [27-30]. Furthermore, to date there are no biomarkers that can predict which patients may obtain most benefit from bevacizumab. Our study identified a group of patients of worse prognosis who benefited greatly from more aggressive treatments, including combination chemotherapy and monochemotherapy plus bevacizumab (both compared with single-agent chemotherapy). The main limitations of our analysis are the lack of randomized comparisons, which implies potential confounding factors, and the limited number of patients. Nevertheless, to our knowledge, this is the first study exploring the role of CTCs as a predictive marker in untreated MBC patients. We believe that our novel findings can serve as a hypothesis generator, supporting the utility to test CTCs as a predictive tool in larger randomized trials.

## Conclusions

In conclusion, our data confirm the prognostic value of CTC enumeration and provide evidence that CTCs can be studied as a unique model to develop tailored treatments for MBC patients.

## Abbreviations

CI: confidence interval; CTC: circulating tumor cell; HER-2: human epidermal growth factor receptor-2; MBC: metastatic breast cancer; OS: overall survival; PFS: progression-free survival.

## Competing interests

The authors declare that they have no competing interests.

## Authors' contributions

MG conceived and designed the study, collected clinical data, performed data analysis and interpretation, and drafted the manuscript. AG collected clinical data, performed data analysis and interpretation, and drafted the manuscript. SJ performed the CellSearch^® ^CTC analysis. KRH performed data analysis and interpretation. UDG and MM collected clinical data. BCH reviewed CTC images and reviewed the manuscript. NTU, RHA and VV provided clinical assessment of patients and reviewed the manuscript. GNH conceived and designed the study, and reviewed the manuscript. MDL and SDP performed data analysis and reviewed the manuscript. JMR conceived and designed the study, and reviewed the manuscript. MC conceived and designed the study, performed data analysis and interpretation, and reviewed the manuscript. All authors read and approved the final version of the manuscript.
